# Effect of levosimendan on the contractility of muscle fibers from nemaline myopathy patients with mutations in the nebulin gene

**DOI:** 10.1186/s13395-015-0037-7

**Published:** 2015-04-28

**Authors:** Josine M de Winter, Barbara Joureau, Vasco Sequeira, Nigel F Clarke, Jolanda van der Velden, Ger JM Stienen, Henk Granzier, Alan H Beggs, Coen AC Ottenheijm

**Affiliations:** Department of Physiology, Institute for Cardiovascular Research, VU University Medical Center Amsterdam, De Boelelaan 1118, 1081, BT Amsterdam, The Netherlands; INMR, The Children’s Hospital at Westmead and Discipline of Paediatrics & Child Health, University of Sydney, Cnr Hawkesbury Road & Hainsworth Street, Sydney, Australia; Department of Physics and Astronomy, Faculty of Science, VU University, De Boelelaan 1105, Amsterdam, The Netherlands; Department of Cellular and Molecular Medicine, University of Arizona, 1333 N. Martin Avenue, Tucson, USA; Division of Genetics and Genomics, The Manton Center for Orphan Disease Research, Boston Children’s Hospital, Harvard Medical School, 25 Shattuck Street, Boston, USA

**Keywords:** Nemaline myopathy, Nebulin, Levosimendan, Calcium-sensitizer, Muscle force, Muscle mechanics

## Abstract

**Background:**

Nemaline myopathy (NM), the most common non-dystrophic congenital myopathy, is characterized by generalized skeletal muscle weakness, often from birth. To date, no therapy exists that enhances the contractile strength of muscles of NM patients. Mutations in *NEB*, encoding the giant protein nebulin, are the most common cause of NM. The pathophysiology of muscle weakness in NM patients with *NEB* mutations (*NEB*-NM) includes a lower calcium-sensitivity of force generation. We propose that the lower calcium-sensitivity of force generation in *NEB*-NM offers a therapeutic target. Levosimendan is a calcium sensitizer that is approved for use in humans and has been developed to target cardiac muscle fibers. It exerts its effect through binding to slow skeletal/cardiac troponin C. As slow skeletal/cardiac troponin C is also the dominant troponin C isoform in *slow-twitch skeletal* muscle fibers, we hypothesized that levosimendan improves slow-twitch muscle fiber strength at submaximal levels of activation in patients with *NEB*-NM.

**Methods:**

To test whether levosimendan affects force production, permeabilized slow-twitch muscle fibers isolated from biopsies of *NEB*-NM patients and controls were exposed to levosimendan and the force response was measured.

**Results:**

No effect of levosimendan on muscle fiber force in *NEB*-NM and control skeletal muscle fibers was found, both at a submaximal calcium level using incremental levosimendan concentrations, and at incremental calcium concentrations in the presence of levosimendan. In contrast, levosimendan did significantly increase the calcium-sensitivity of force in human single cardiomyocytes. Protein analysis confirmed that the slow skeletal/cardiac troponin C isoform was present in the skeletal muscle fibers tested.

**Conclusions:**

These findings indicate that levosimendan does not improve the contractility in human skeletal muscle fibers, and do not provide rationale for using levosimendan as a therapeutic to restore muscle weakness in *NEB*-NM patients. We stress the importance of searching for compounds that improve the calcium-sensitivity of force generation of slow-twitch muscle fibers. Such compounds provide an appealing approach to restore muscle force in patients with *NEB*-NM, and also in patients with other neuromuscular disorders.

**Electronic supplementary material:**

The online version of this article (doi:10.1186/s13395-015-0037-7) contains supplementary material, which is available to authorized users.

## Background

Nemaline myopathy (NM) is the most common non-dystrophic congenital myopathy (incidence approximately 1:50,000) [1]. Hallmark features of NM include muscle weakness and the presence of nemaline bodies in muscle fibers [2]. To date, ten genes have been implicated in NM. Seven of these genes encode proteins of the skeletal muscle thin filament (alpha-tropomyosin-3 and beta-tropomyosin (*TPM3* and *TPM2*), nebulin (*NEB*), actin alpha 1 (*ACTA1*), troponin T type 1 (*TNNT1*), cofilin-2 (*CFL2)*), and leiomodin-3 (*LMOD3*) [3,4], and three genes encode kelch domain proteins that are associated with thin filament proteins and may be involved in regulating thin filament protein stability or turnover (kelch repeat and BTB (POZ) Domain Containing 13 (*KBTBD13*), (kelch-like family member 40 (*KLHL40*) and kelch-like family member 41 (*KLHL41*)) [5-11].

Mutations in *NEB* are the most common cause of NM, accounting for more than 50% of NM cases [12,13]. Nebulin is a giant sarcomeric protein (approximately 800 kDa), and a single nebulin molecule spans nearly the entire length of the thin filament. Previous studies of a nebulin knockout mouse model showed that nebulin stabilizes the thin filament and specifies its length [14-18]. Evidence also suggests that nebulin modulates both the kinetics of actomyosin cross-bridge formation [19,20] and the calcium-sensitivity of thin filament activation [20,21]. Recent work revealed that skeletal muscle fibers of NM patients with *NEB* mutations (*NEB*-NM) develop muscle weakness due to loss of these functions of nebulin; their myofibers contain thin filaments of shorter length, they show altered actomyosin cross-bridge kinetics [22-24], and they have a lower calcium-sensitivity of force generation [23].

To date, no therapy exists that enhances force generation in *NEB*-NM. We propose that the lower calcium sensitivity of force generation in *NEB*-NM offers a therapeutic target. Recent work from our group addressed the ability of a fast skeletal muscle troponin activator to enhance the calcium sensitivity of force generation in skeletal muscle fibers from *NEB*-NM patients. These troponin activators target fast-twitch muscle fibers [25], and it was demonstrated that the compound increased the calcium sensitivity of force generation in fast-twitch fibers of *NEB*-NM patients [26]. As human skeletal muscles consist of a mixture of fast- and slow-twitch muscle fibers, it would be desirable to also target the calcium sensitivity of slow-twitch muscle fibers in NM patients, especially as these patients have been shown to have a predominance of slow-twitch muscle fibers [2]. Levosimendan is a calcium sensitizer that is approved for use in humans and has been developed to target cardiac muscle fibers. It exerts its effect through binding to slow skeletal/cardiac troponin C and improves cardiac contractility *in vivo* [27] and *in vitro* [28]. Slow skeletal/cardiac troponin C is also the dominant troponin C isoform in *slow-twitch skeletal* muscle fibers, and recent work suggested that levosimendan improves submaximal contractility of slow-twitch muscle fibers in the diaphragm of animal models and in humans [29-31].

Based on these findings, we hypothesized that levosimendan improves slow-twitch muscle fiber strength at submaximal levels of activation in patients with *NEB*-NM. To test this hypothesis, we exposed muscle fibers isolated from biopsies of *NEB*-NM patients to levosimendan and measured the force response. Our findings suggest that levosimendan does not improve the *in vitro* contractility of slow-twitch muscle fibers at submaximal activation levels in patients with *NEB-*NM.

## Methods

### Skeletal muscle biopsies of *NEB*-NM patients

Quadriceps muscle specimens, remaining from diagnostic procedures or obtained during clinically indicated surgical procedures, were collected from four NM patients with confirmed *NEB* gene mutations and from three adult control subjects with no medical history. Ethical approval for study of the NM biopsies was granted by the Institutional Review Board of Boston Children’s Hospital, and these biopsies were obtained from discarded clinical specimens following informed consent under protocol 03-08-128R. Details on the clinical and genetic data of the subjects have been published previously (biopsy IDs T33, T1033, T1069, and T887 [26]).

The three adult control muscle biopsies were obtained under supervision of the HREC, Children’s Hospital at Westmead (CHW/10/45). All biopsies were stored frozen and unfixed at −80°C until use.

### Skeletal muscle mechanics

Small strips dissected from the muscle biopsies were permeabilized overnight as described previously [24]. This procedure renders the membranous structures in the muscle fibers permeable, which enables activation of the myofilaments with exogenous calcium. Preparations were washed thoroughly with relaxing solution and stored in 50% glycerol/relaxing solution at −20°C. Small muscle bundles (cross-sectional area approximately 0.07 mm^2^, *NEB*-NM patients) and single muscle fibers (control subjects) were dissected from the permeabilized strips and were mounted using aluminum T-clips between a length motor (ASI 403A, Aurora Scientific Inc., Ontario, Canada) and a force transducer element (ASI 315C-I, Aurora Scientific Inc., Ontario, Canada) in a single fiber apparatus (ASI 802D, Aurora Scientific Inc., Ontario, Canada) that was mounted on the stage of an inverted microscope (Zeiss Axio Observer A1, Zeiss, Thornwood, NY, USA). Sarcomere length was set using a high-speed VSL camera and ASI 900B software (Aurora Scientific Inc., Ontario, Canada). Mechanical experiments were performed at a sarcomere length of 2.1 μm, a length selected to minimize force differences due to shorter thin filaments in fibers from *NEB*-NM patients [24]. Fiber width and diameter were measured at three points along the fiber, and the cross-sectional area was determined assuming an elliptical cross section. Three different types of bathing solutions were used during the experimental protocols: a relaxing solution (100 mM BES; 6.97 mM EGTA; 6.48 mM MgCl_2_; 5.89 mM Na_2_-ATP; 40.76 mM K-propionate; 14.5 mM creatine phosphate), a pre-activating solution with low EGTA concentration (100 mM BES; 0.1 mM EGTA; 6.42 mM MgCl_2_; 5.87 mM Na_2_-ATP; 41.14 mM K-propionate; 14.5 mM creatine phosphate; 6.9 mM HDTA), and an activating solution (100 mM BES; 7.0 mM Ca-EGTA; 6.28 mM MgCl_2_; 5.97 mM Na_2_-ATP; 40.64 mM K-propionate; 14.5 mM creatine phosphate). The temperature of the bathing solutions was kept constant at 20°C using a TEC controller (ASI 825A, Aurora Scientific Inc. Ontario, Canada).

Concentrated stock solutions of levosimendan (a kind gift from Orion Pharma, Espoo, Finland) were prepared in dimethyl sulfoxide (DMSO). Before use, levosimendan stock solutions were diluted with experimental solutions. The final concentration of DMSO did not exceed 0.03%. Control experimental solutions contained 0.03% DMSO (vehicle). To determine a dose-response curve for levosimendan in muscle fibers from controls (CTRL) and from *NEB*-NM patients, tissue was exposed to an activating solution with a pCa of 5.8 (CTRL) or pCa 5.6 *(NEB*-NM) - these pCas yielded approximately 40% of maximal active tension - with vehicle (to establish the submaximal force generation at that calcium concentration). Subsequently, tissue was exposed to a similar activating solution, but which contained increasing concentrations of levosimendan (0.1, 1, 10, 25, and 100 μM). After exposure to the final levosimendan concentration, the fibers were once more activated in vehicle to rule out rundown of force. Note that 0.03% DMSO did not affect muscle fiber contractility (data not shown).

Finally, to determine the effect of levosimendan (100 μM) on the force-pCa relation, permeabilized muscle fiber bundles or single fibers were sequentially bathed in a relaxing solution, a pre-activation solution, and solutions with pCa values ranging from 9.0 to 4.5 - all containing 100 μM levosimendan - and the steady-state force was measured. Measured force values were normalized to the maximal force obtained at pCa 4.5. The obtained force-pCa data were fit to the Hill equation (Y = 1/(1 + 10n_H_(pCa − pCa_50_))) where the pCa_50_ corresponds to the calcium concentration that yields half-maximal force and the Hill coefficient, n_H_, to myofilament cooperativity [32].

### Myocardial human biopsies

Human left ventricular tissue was obtained from three non-failing donors of whom the myocardium was not required for transplantation. Donors had no history of cardiac abnormalities, normal ECG, and normal ventricular function on echocardiography within 24 h of heart transplantation. Tissue was collected in cardioplegic solution and immediately frozen and stored in liquid nitrogen. Samples were obtained after written informed consent and with approval of the local Human Research Ethics Committee of the University of Sydney (#7326). The investigation conforms with the principles outlined in the Declaration of Helsinki (1997).

### Cardiomyocyte mechanics

Cardiac tissue samples were thawed in relaxing solution (5.95 mM Na_2_-ATP, 6.04 mM MgCl_2_, 2 mM EGTA, 139.6 mM KCl, 10 mM Imidazole, pH 7.0), and cardiomyocytes were mechanically isolated by tissue disruption. Thereafter, cardiomyocytes were chemically permeabilized by incubation for 5 min in relaxing solution containing 0.5% (*v*/*v*) Triton-X100 and glued between a force transducer and a piezoelectric motor as described previously [33]. Isometric force measurements were performed at a submaximal calcium concentration (pCa 5.6), which generates 44% ± 5% of maximal force at saturating calcium levels (pCa 4.5). Sarcomere length was adjusted to 1.8 μm and determined by means of a spatial Fourier transformation as described previously [33].

Levosimendan was dissolved in DMSO and diluted in pCa 5.6 calcium batches to a final concentration ranging from 0.1 to 100 μM. The final DMSO concentration did not exceed 0.03%. To control for artificial side effects resulting from DMSO, the latter was diluted in pCa 5.6 (DMSO final concentration <0.03%) and served as control, herein termed as vehicle. No difference in developed force was found between pCa 5.6 compared with vehicle up to the maximum concentration levosimendan used.

### Myosin heavy chain composition

A specialized sodium dodecyl sulfate polyacrylamide gel electrophoresis (SDS-PAGE) was used to determine the myosin heavy chain isoform composition of the muscle fiber preparations that we used in our contractility experiments [24]. In brief, muscles fibers were denatured by boiling for 2 min in SDS sample buffer. The stacking gel contained a 4% acrylamide concentration (pH 6.7), and the separating gel contained 7% acrylamide (pH 8.7) with 30% glycerol (*v*/*v*). The gels were run for 24 h at 15°C and a constant voltage of 275 V. Finally, the gels were silver-stained, scanned, and analyzed with One-D scan EX (Scanalytics Inc., Rockville, MD, USA) software.

### Troponin C Western blotting

Muscle samples were solubilized as described previously [34]. In brief, frozen muscle samples were homogenized in a liquid nitrogen cooled mortar and re-suspended in 1 ml cold 10% trichloracetic acid (TCA) solution dissolved in acetone containing dithiothreitol (DTT) (0.2% *w*/*v*) and stored at −80°C for 60 min. Subsequently, homogenates were brought to room temperature stepwise: 20 min at −20°C, 20 min at 4°C, and 20 min at room temperature (while being mixed on a vortex in between all steps). Then, muscle homogenates were centrifuged at 12,000*g* for 15 min followed by washing the tissue pellets with 1 ml of 0.2% *w*/*v* DTT-acetone solution and shaking them for 5 min at room temperature. This cycle of centrifugation, washing, and shaking was repeated five times. Tissue pellets were freeze-dried overnight and homogenized in sample buffer containing 15% glycerol, 62.5 mM Tris (pH 6.8), 1% *w*/*v* SDS, and 2% *w*/*v* DTT (final concentration 5 μg dry weight/μl).

To discriminate between both slow skeletal/cardiac troponin C and fast skeletal troponin C isoforms, 4 μl of muscle homogenates in 1-D sample buffer (20 μg of dry weight tissue) were loaded on a 15% acrylamide SDS-PAGE gel. Subsequently, the gel ran first for 20 min at 200 V and thereafter 160 min at 400 V at 15°C. After completion of the run, the gel was blotted for 90 min at constant amperage of 320 mA using a semidry blotting system (Trans-Blot SD Cell, Bio-Rad, Hercules, CA, USA). After staying overnight in blocking solution (5% milk in TBS-T), the blot was incubated at room temperature for 90 min in a pan-specific antibody directed against troponin C (#4002, Cell Signaling, Boston, MA, USA) (1:500 in blocking solution), washed for 30 min and put in secondary antibody goat anti-rabbit HRP (Dako, Glostrup, Denmark) (1:2,500 in blocking solution) for 60 min at room temperature. Thereafter, the blot was washed with TBS-T for 30 min and treated with ECL prima reagents (GE Healthcare, Buckinghamshire, UK) for 5 min. The blot was scanned using a LAS 3000 (Fujifilm Medical Systems, Stamford, CT, USA).

### Statistical analyses

Data are presented as mean ± SEM. For statistical analyses, *t* test, one-way ANOVA, and two-way ANOVA were used. For dose-response tests, one-way repeated measures ANOVA was used, with Dunnett’s Multiple Comparison Test as *post hoc* test. *P* < 0.05 was considered to be statistically significant.

## Results

### The contractile performance of permeabilized muscle fibers from nemaline myopathy patients with nebulin mutations

#### Muscle contractility experiments

**Figure 1:**
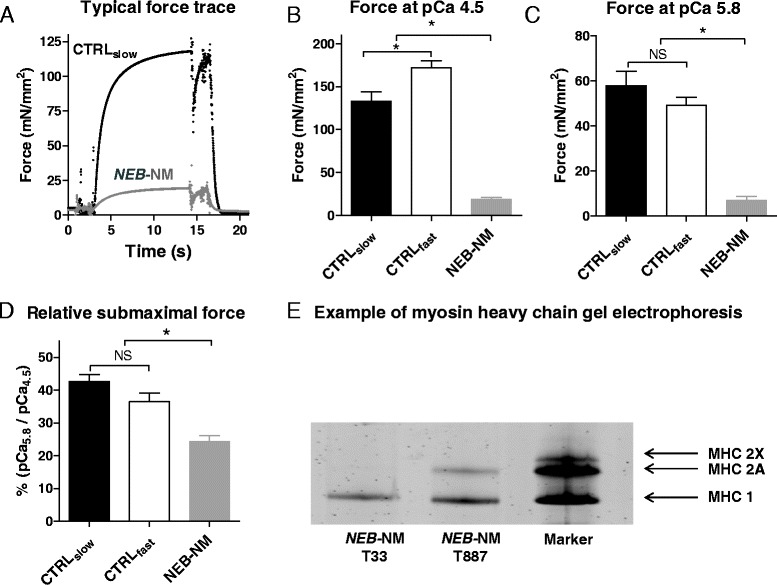
Typical force traces, maximal tension, submaximal tension, relative submaximal tension, and specialized SDS-PAGE gel example. **(A)** Typical force trace of a slow-twitch single muscle fiber of a control subject and of a muscle fiber bundle of a *NEB*-NM patient (biopsy ID T33 is shown) during exposure to an experimental solution with pCa 4.5. Note the severely reduced maximal tension of the muscle fibers of the *NEB*-NM patient. **(B)** Maximal tension (pCa 4.5) of muscle fibers of healthy controls and *NEB*-NM patients. **(C)** Submaximal tension (pCa 5.8) of muscle fibers of healthy controls and *NEB*-NM patients. **(D)** Relative submaximal tension (pCa 5.8/4.5). Note that the relative submaximal tension of muscle fibers of *NEB*-NM patients is reduced, indicating a reduced calcium sensitivity of force. **(E)** Example of a specialized SDS-PAGE gel that was used to determine the myosin heavy chain isoform composition of the muscle fiber preparations that we used in our contractility experiments. Here, the bundle from *NEB-*NM T33 is composed of type 1 myosin heavy chain isoforms (MHC 1), the bundle from *NEB*-NM T887 contains both MHC 1 and type 2A myosin heavy chain isoforms (MHC 2A), and the marker exhibits both MHC 1 and MHC 2A as myosin 2X isoforms (MHC 2X). *NEB*-NM, nemaline myopathy patients with mutations in the nebulin gene; NS, not significant. *P < 0.05.

The force-generating capacity of permeabilized muscle fiber bundles isolated from nemaline myopathy biopsies with mutations in the nebulin gene (*NEB*-NM) was lower compared to muscle fibers from healthy controls at both maximal *and* submaximal calcium levels (for typical force traces see Figure 1A). At pCa 4.5 - corresponding to a calcium concentration that yielded maximal force generation - *NEB*-NM muscle fiber bundles revealed a significant lower force-generating capacity (19 ± 3 mN/mm^2^) compared to slow-twitch single fibers from control biopsies (CTRL_slow_) (133 ± 11 mN/mm^2^) and fast-twitch single fibers from control biopsies (CTRL_fast_) (172 ± 8 mN/mm^2^) (Figure 1B). In addition, at submaximal calcium levels (pCa 5.8), *NEB*-NM muscle fibers (7 ± 2 mN/mm^2^) were significantly weaker than CTRL_slow_ (58 ± 6 mN/mm^2^) and CTRL_fast_ fibers (49 ± 4 mN/mm^2^) (Figure 1C).

Interestingly, when expressing the force generating capacity of the muscle fibers at a submaximal calcium concentration (pCa 5.8) as a percentage of the maximal force-generating capacity (pCa 4.5, Fmax), *NEB*-NM muscle fibers reveal a significant lower relative contractile performance at submaximal calcium levels (24%Fmax ± 2%Fmax) compared to CTRL_slow_ (43%Fmax ± 2%Fmax) and CTRL_fast_ fibers (37%Fmax ± 3%Fmax) (Figure 1D). This observation indicates that *NEB-*NM muscle fibers have a lower calcium sensitivity of force than fibers from controls.

#### Myosin heavy chain composition

As the force-generating capacity of muscle fibers depends on the myosin heavy chain isoform composition, we determined the relative abundance of slow myosin isoforms (MHC_slow_) and fast myosin isoforms MHC 2A and 2× (MHC_fast_) in the single fibers of control subjects and the muscle fiber bundles of *NEB*-NM patients that were used for the contractility experiments. Our analyses showed that *NEB*-NM muscle bundles were composed of 75% ± 6% MHC_slow_ and of 25% ± 6% MHC_fast_ (Figure 1E). Thus, the biopsies of the *NEB-*NM patients contained predominantly slow-twitch muscle fibers.

### The effect of levosimendan on force in permeabilized skeletal muscle fibers

#### Dose-response relation

**Figure 2:**
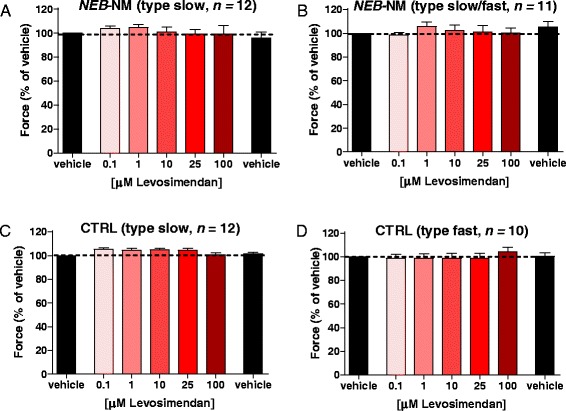
The effect of incremental concentrations of levosimendan on submaximal force (approximately 40% of maximal force) of *NEB*-NM slow-twitch **(A)** and mixed **(B)** muscle fibers. Prior to and directly after exposure to the levosimendan concentrations, fibers were exposed to vehicle to rule out confounding effects of force rundown during the protocol. Note that levosimendan did not increase force compared to vehicle. Similar results were observed in slow-twitch **(C)** and fast-twitch **(D)** muscle fibers of control subjects. *NEB*-NM, nemaline myopathy patients with mutations in the nebulin gene; CTRL, control.

We tested levosimendan at a range of concentrations and at a pCa that yielded approximately 40% of maximal force in both *NEB*-NM fibers and CTRL fibers. In both *NEB-*NM muscle bundles that expressed solely MHC_slow_ and *NEB*-NM muscle bundles that expressed a mix of MHC_slow_ and MHC_fast_, no effect of levosimendan on force was observed at any of the concentrations tested (Figure 2A,B). Muscle of these *NEB*-NM patients is nebulin-deficient (as reported previously [26]). To verify whether an effect of levosimendan on force was blunted by nebulin deficiency of *NEB*-NM muscle fibers, we also performed dose-response experiments on CTRL_slow_ and CTRL_fast_ muscle fibers at approximately 40%Fmax. Again, it was observed that none of the concentrations tested an effect of levosimendan on muscle fiber force (Figure 2C,D).

#### Force-pCa curve

**Figure 3:**
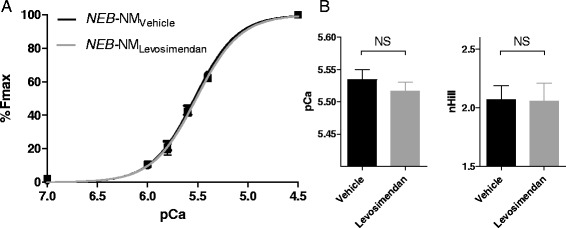
No shift of the force-pCa curve was observed upon exposure to 100 μM levosimendan. **(A)** Levosimendan (100 μM) did not affect the force-pCa relation of muscle fibers of *NEB*-NM patients, as reflected by the unaltered pCa_50_ **(B, left panel)** and nHill **(B, right panel)**. *NEB*-NM, nemaline myopathy patients with mutations in the nebulin gene; NS, not significant.

The dose-response experiments were performed at a single pCa, and therefore we also studied the effect of levosimendan on fiber force at a range of calcium concentrations. In *NEB*-NM muscle fibers, no shift of the force-pCa curve was observed upon exposure to 100 μM levosimendan (Figure 3A,B), as reflected by no change in the pCa_50_ value (5.52 ± 0.01 *vs*. 5.53 ± 0.02, levosimendan in vehicle *vs*. vehicle alone, respectively) and no change in n_H_, a measure of cooperativity of activation (2.06 ± 0.16 *vs*. 2.07 ± 0.12, levosimendan in vehicle *vs*. vehicle alone, respectively).

Thus, we observed no effect of levosimendan on muscle fiber force in *NEB*-NM and CTRL skeletal muscle fibers, neither at a submaximal calcium level using incremental levosimendan concentrations nor at incremental calcium concentrations in the presence of levosimendan.

### The effect of levosimendan on force in human cardiomyocytes

**Figure 4:**
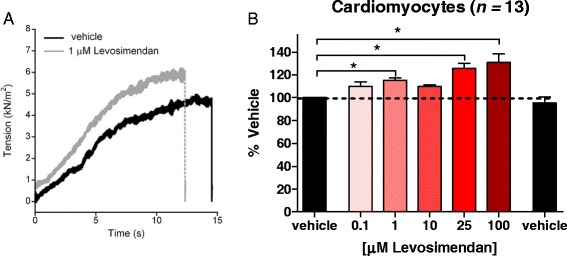
Typical force trace and the effect of incremental concentrations of levosimendan on submaximal force. **(A)** Typical force trace of a human cardiomyocyte. Force increases while exposing the cardiomyocyte to an experimental solution with submaximal exogenous calcium levels (pCa 6.5). Relaxation is induced by exposing the cardiomyocyte to an experimental solution with a very low calcium concentration (pCa 9.0). **(B)** The effect of incremental concentrations of levosimendan on submaximal force (approximately 40% of maximal force) of human cardiomyocytes. Prior to and directly after exposure to the levosimendan concentrations, cardiomyocytes were exposed to vehicle to rule out confounding effects of force rundown during the protocol. Note that at various concentrations (1, 25, and 100 μM) levosimendan significantly increased submaximal force. *P < 0.05.

As previous work showed that levosimendan augments submaximal force in cardiomyocytes [28], we next studied whether - in our hands - levosimendan affects submaximal force in cardiomyocytes (see Figure 4A for typical force traces). These experiments revealed a significant dose-dependent increase of submaximal force in human cardiomyocytes at levosimendan concentrations of 1 μM (115% ± 2% of vehicle), 25 μM (125% ± 4% of vehicle), and 100 μM (131% ± 8% of vehicle) (Figure 4B). Thus, we confirmed the efficacy of levosimendan in human cardiomyocytes.

### Slow skeletal/cardiac troponin C levels in skeletal muscle fibers of *NEB*-NM patients

**Figure 5:**
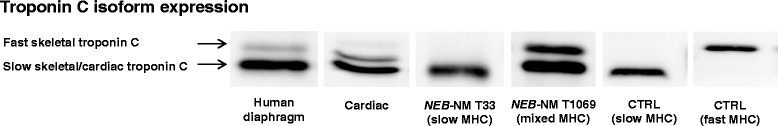
Western blot example, with anti-TnC antibody, including muscle homogenates of human diaphragm muscle (which is known to contain both slow skeletal/cardiac troponin C and fast skeletal troponin C), human left ventricle (which also contains slow skeletal/cardiac troponin C), and quadriceps muscle of *NEB*-NM patients (biopsy IDs T33 and T1069 are shown) and healthy controls. Note that slow skeletal/cardiac troponin C is abundant in *NEB*-NM muscle. *NEB*-NM, nemaline myopathy patients with mutations in the nebulin gene; CTRL, control; MHC, myosin heavy chain.

Levosimendan exerts its calcium-sensitizing effect by binding to slow skeletal/cardiac troponin C. As we observed no effect of levosimendan in slow-twitch skeletal muscle fibers, we tested whether these fibers indeed expressed slow skeletal/cardiac troponin C isoforms. Figure 5 shows that *NEB*-NM_slow_ muscle fibers, *NEB*-NM_mixed_ muscle fibers, and CTRL_slow_ muscle fibers express the slow skeletal/cardiac troponin C isoform. Thus, slow skeletal/cardiac troponin C, the isoform through which levosimendan exerts its effect, was abundantly expressed in *NEB*-NM and CTRL_slow_ muscle samples.

## Discussion

A reduced calcium sensitivity of force generation contributes to muscle weakness in *NEB*-NM muscle. Therefore, in the present study, we aimed to test the ability of levosimendan, the only calcium sensitizer approved for use in humans, to improve the calcium sensitivity of force generation in slow-twitch muscle fibers of *NEB*-NM patients. Our findings indicate that levosimendan does not improve the calcium sensitivity of force generation of slow-twitch muscle fibers isolated from biopsies of *NEB*-NM patients.

### Submaximal force generation is lower in muscle fibers from *NEB-*NM patients compared to that in muscle fibers from controls

The severe reductions in force levels generated by fibers of *NEB*-NM patients are caused by contractile deficits due to defects in sarcomeric proteins [35], rather than lower neural activation, ineffective excitation-contraction coupling, or other non-contractile defects. Mechanisms that contribute to weakness of muscle fibers in *NEB*-NM include myofibrillar disarray [3,22], shorter thin filaments [24], altered cross-bridge cycling kinetics [19,20], and a reduced calcium sensitivity of force generation [20,21].

The *NEB*-NM samples studied here were from the same cohort as studied recently by de Winter and coworkers [26]. In that study, it was shown that muscle fibers of *NEB*-NM patients have a lower calcium sensitivity of force generation compared to fibers from control subjects [26]. Here, we confirmed these findings on freshly isolated fibers from biopsies of these patients. Figure 1 illustrates that at pCa 5.8 - a pCa that yields submaximal force - the force generated, relative to maximal force, is lower in *NEB*-NM fiber bundles when compared to fibers from control subjects. As discussed previously, the lower calcium sensitivity of force in *NEB*-NM fibers is likely to be caused, at least in part, by nebulin deficiency [20,21]. Note that previous work indicated that muscle fibers in the biopsies of these patients contained approximately 25% of normal nebulin levels [26].

### Levosimendan does not improve the submaximal force of *NEB*-NM muscle fibers

Targeting the reduced calcium sensitivity of force is an appealing approach to combat muscle weakness in *NEB*-NM patients. Recently, we tested the ability of a fast skeletal troponin activator to restore force of fast-twitch muscle fibers of NM patients [26] and of a mouse model for *NEB*-NM [21]. Fast skeletal troponin activators selectively sensitize the sarcomeres of fast-twitch skeletal muscle fibers to calcium ions by increasing the affinity of fast skeletal troponin C to calcium [25]. As neuromuscular input results in calcium release in the muscle, the increased calcium sensitivity will enhance submaximal muscle strength*.* We have shown that fast skeletal troponin activators augment the *in vitro* contractile strength of muscle fibers of *NEB*-NM patients and *NEB*-NM mice; the calcium sensitivity of force generation even increased to levels higher than that observed in healthy, untreated, control muscle [21,26].

Skeletal muscles are, however, composed of a mixture of fast- and slow-twitch fibers, and their proportion varies between muscles. For instance, in healthy humans, the quadriceps muscle contains approximately 50% slow-twitch fibers, while in the soleus muscle more than 80% of fibers are of the slow-twitch type. During activation of an individual muscle, first the motor units that comprise of slow-twitch muscle fibers are the first ones recruited. Subsequently, and only if required, the larger motor units that consist of fast-twitch fibers are recruited. In addition, NM patients typically exhibit a shift towards a larger proportion of slow-twitch fibers [2]. These factors highlight the therapeutic opportunity of improving the calcium sensitivity of force of *slow-twitch* muscle fibers in *NEB*-NM patients.

Levosimendan was developed to increase the calcium sensitivity of cardiac muscle to enhance contractility in the failing heart [28,36]. It binds to slow skeletal/cardiac troponin C, encoded by the *TNNC1* gene, and stabilizes the conformation of the troponin complex [36]. Slow skeletal/cardiac troponin is also the dominant troponin C isoform in slow-twitch *skeletal* muscle fibers, suggesting that levosimendan could also improve the calcium sensitivity of slow-twitch muscle fibers. Indeed, recent work showed that slow-twitch diaphragm muscle fibers of patients with chronic obstructive pulmonary disease had an increased submaximal force after exposure to levosimendan [31]. Furthermore, *in vivo* administration of levosimendan improved diaphragm contractility in healthy subjects [29]. More specifically, the latter study showed that levosimendan improved the neuromechanical efficiency and reduced the development of fatigue of the human diaphragm during loading tasks *in vivo* [29]. These findings highlight that the potential benefit of calcium sensitizers includes not only increased force development but also increased efficiency by reducing the amount of cytosolic calcium that is required to generate a given level of force. The energy utilization of SERCA accounts for 30% to 40% of total ATP consumption during muscle contraction [37,38]. Therefore, the use of a calcium sensitizer has the potential to reduce the amount of calcium that cycles each contraction and thereby reduce muscle fatigue. This might be especially beneficial for the respiratory muscles in patients with NM, considering that respiratory failure due to diaphragm weakness is the main cause of death in neonates with NM [2].

These previous findings with levosimendan provided an impetus to study its effect on slow-twitch muscle fibers of *NEB*-NM fibers. However, to our surprise, we observed no effect of levosimendan on submaximal force of slow-twitch muscle fibers of *NEB*-NM patients (Figures 2 and 3). Similarly, slow-twitch fibers of control subjects showed no response to levosimendan. Note that we also tested levosimendan in slow-twitch human *diaphragm* muscle fibers - the muscle type that revealed positive results in previous studies [30,31] - but we observed no effect on the calcium sensitivity of force generation (see Additional file 1: Figure S1). It should be noted that the lack of a response to levosimendan was not caused by ‘defective’ levosimendan. We ruled out this possibility by testing the effect of levosimendan on human cardiomyocytes, and in line with extensive previous work (for instance [28,39,40]), we observed a significant increase in the calcium sensitivity of force after exposure to levosimendan (Figure 4). Furthermore, we evaluated whether slow-twitch muscle fibers of control and *NEB*-NM biopsies contained slow skeletal/cardiac troponin C isoforms. As shown in Figure 5, these fibers did indeed contain slow skeletal/cardiac troponin C. Thus, the absence of a response to levosimendan in slow-twitch muscle fibers was not caused by deficiency of slow skeletal/cardiac troponin C. Unfortunately, we cannot provide a plausible explanation for the discrepancy between our findings and those from previous studies that did show a response of slow-twitch fibers to levosimendan [30,31].

## Conclusions

In conclusion, the *ex vivo* findings with isolated muscle fibers do not provide rationale for levosimendan as a therapeutic to restore muscle weakness in *NEB*-NM patients. Future studies, to be performed by independent laboratories, should confirm these findings. We stress the importance of searching for compounds that improve the calcium sensitivity of force of slow-twitch muscle fibers. For instance, future research could test the efficacy of, not yet registered, compounds such as EMD57033 and pimobendan [41]. These compounds may be able to restore muscle force not only in patients with *NEB*-NM but also in patients with other neuromuscular disorders.

## Additional file

Additional file 1: Figure S1. Levosimendan in human diaphragm muscle fibers. The effect of incremental concentrations of levosimendan on submaximal force (pCa 5.8, which yielded approximately 40% of maximal force) of individual slow-twitch fibers from human diaphragm (*n* = 7). Dose-force response experiments were performed according to the protocol reported in the ‘Methods’ section. Single muscle fibers were isolated from a diaphragm muscle biopsy that was obtained from a patient that underwent resection of an early lung malignancy (male, age 64). The biopsy protocol was approved by the institutional review board at VU University Medical Center Amsterdam (#2010/69). Written informed consent was obtained from the patient.

## Abbreviations

*ACTA1*: actin alpha 1
ANOVA: analysis of variance
ATP: adenosine triphosphate
BES: N,N-bis-(2-hydroxyethyl)-2-aminoethanesulfonic acid
BTB: bric-a-brac, tramtrac, and broad complex
Ca-EGTA: calcium-ethylene glycol tetraacetic acid
*CFL2*: cofilin-2
CTRL_fast_: muscle fibers from controls that exhibit myosin heavy chain type 2A and/or type 2X isoforms
CTRL_slow_: muscle fibers from controls that exhibit myosin heavy chain type 1 isoforms
DMSO: dimethyl sulfoxide
DTT: dithiothreitol
ECG: electrocardiogram
ECL: enhanced chemiluminescence
EGTA: ethylene glycol tetraacetic acid
Fmax: maximal force generating capacity (normalized to the cross-sectional area of the muscle fiber or bundle)
HDTA: 1,6-diaminohexane NNNN tetra acetic acid
HRP: horseradish peroxidase
*KBTBD13*: kelch repeat and BTB (POZ) Domain Containing 13
KCl: potassium chloride
*KLHL40*: kelch-like family member 40
*KLHL41*: kelch-like family member 41
K-propionate: potassium-propionate
*LMOD3*: leiomodin-3
MgCl_2_: magnesium chloride
MHC 1: myosin heavy chain type 1 isoform
MHC 2A: myosin heavy chain type 2A isoform
MHC 2X: myosin heavy chain type 2X isoform
MHC_fast_: myosin heavy chain type 2A and/or type 2X isoforms
MHC_slow_: myosin heavy chain type 1 isoform
Na_2_-ATP: adenosine 5′-triphosphate disodium salt hydrate
*NEB*: nebulin
*NEB*-NM: nemaline myopathy patients with mutations in the nebulin gene
*NEB*-NM_mixed_: muscle fibers from nemaline myopathy patients with mutations in the nebulin gene that exhibit both myosin heavy chain type 1 isoforms and myosin heavy chain type 2A isoforms
n_H_: the Hill coefficient, a measure of myofilament cooperativity
NM: nemaline myopathy
pCa: negative logarithm of the calcium concentration
pCa_50_: the calcium concentration that yields half-maximal force
POZ: poxvirus and zinc finger
SDS: sodium dodecyl sulfate
SDS-PAGE: sodium dodecyl sulfate polyacrylamide gel electrophoresis
SEM: standard error of the mean
SERCA: sarco/endoplasmic reticulum Ca^2+^-ATPase
TBS-T: tris-buffered saline and tween 20
TCA: trichloracetic acid
TEC: thermoelectric cooler
TNNC1: slow skeletal/cardiac troponin C
*TNNT1*: troponin T type 1
*TPM2*: beta-tropomyosin
*TPM3*: alpha-tropomyosin-3
Tris: tris(hydroxymethyl)aminomethane
