# Supplementary figures and images for: Effect of levosimendan on the contractility of muscle fibers from nemaline myopathy patients with mutations in the nebulin gene
Source: Skelet Muscle. 2015 Apr 28;5:12. doi: 10.1186/s13395-015-0037-7 (PMC4422316; doi:10.1186/s13395-015-0037-7)

## Slide 1
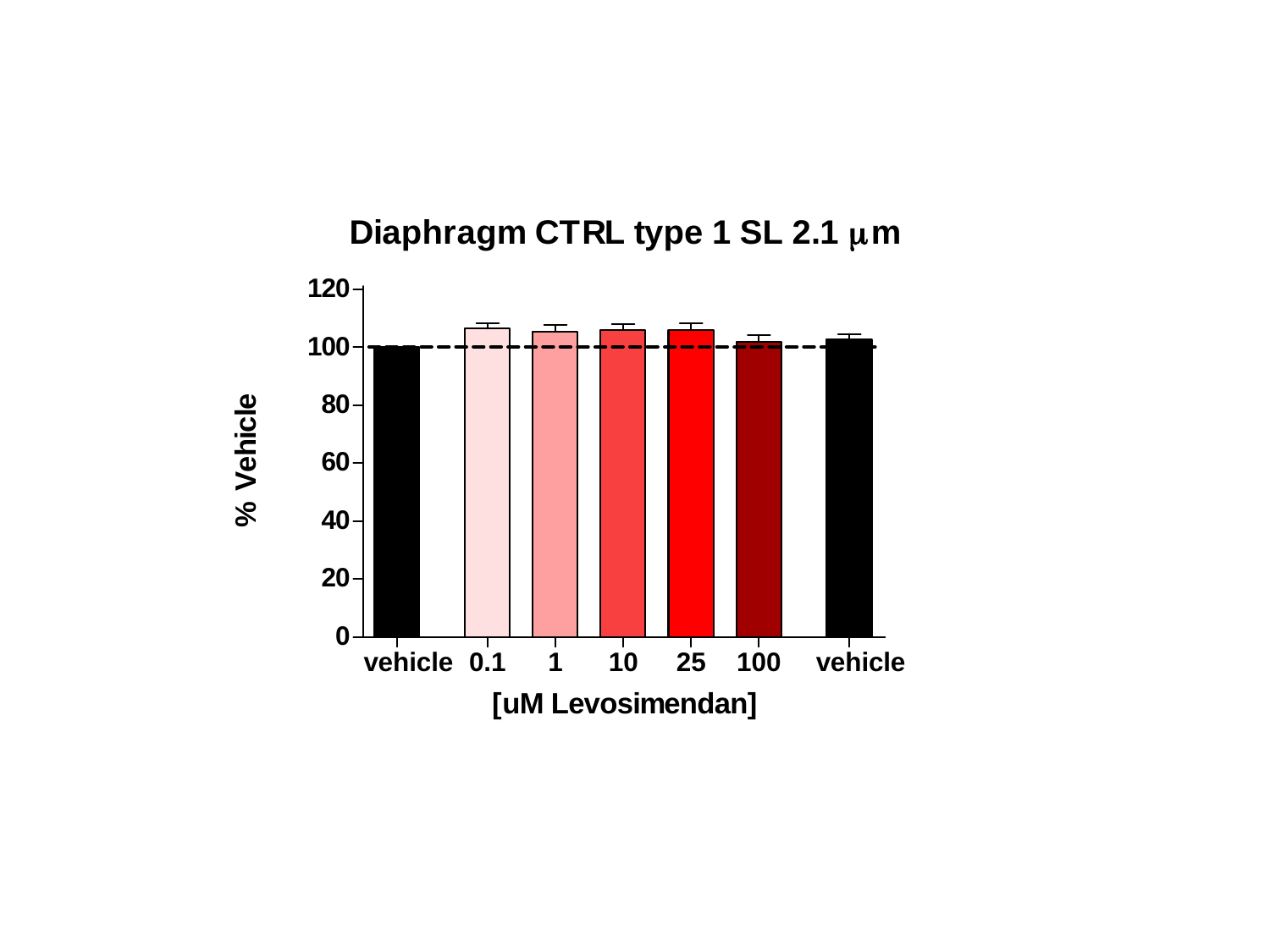

Supplement: Additional file 1: Figure S1. — Levosimendan in human diaphragm muscle fibers. The effect of incremental concentrations of levosimendan on submaximal force (pCa 5.8, which yielded approximately 40% of maximal force) of individual slow-twitch fibers from human diaphragm (n = 7). Dose-force response experiments were performed according to the protocol reported in the ‘Methods’ section. Single muscle fibers were isolated from a diaphragm muscle biopsy that was obtained from a patient that underwent resection of an early lung malignancy (male, age 64). The biopsy protocol was approved by the institutional review board at VU University Medical Center Amsterdam (#2010/69). Written informed consent was obtained from the patient. [file 13395_2015_37_MOESM1_ESM.pptx]
